# *Fusobacterium nucleatum* induces colon anastomosis leak by activating epithelial cells to express MMP9

**DOI:** 10.3389/fmicb.2022.1031882

**Published:** 2022-12-14

**Authors:** Shang Shi, Yang Liu, Zhiyue Wang, Xiangren Jin, Wei Yan, Xiao Guo, Baiqiang Lin, Haoran Wang, Bowen Li, Jianjun Zheng, Yunwei Wei

**Affiliations:** ^1^Oncology and Laparoscopy Surgery, The First Affiliated Hospital of Harbin Medical University, Harbin, China; ^2^Pancreatic and Gastrointestinal Surgery Division, HwaMei Hospital, University of Chinese Academy of Sciences, Ningbo, China; ^3^Ningbo Clinical Research Center for Digestive System Tumors, Ningbo, China; ^4^Imaging Center, HwaMei Hospital, University of Chinese Academy of Sciences, Ningbo, China; ^5^Ningbo Clinical Medical Research Center of Imaging Medicine, Ningbo, China

**Keywords:** *Fusobacterium nucleatum*, anastomotic leak, colorectal cancer, adhesion ability, matrix metalloproteinase 9

## Abstract

**Background:**

Despite advances in anastomotic techniques and perioperative care, the incidence of anastomotic leak (AL) has not substantially decreased over time. Although it is known that AL etiology is multifactorial and the mechanisms involved remain unclear, there is accumulating evidence pointing at AL related to gut microbiota.

**Method:**

We firstly performed a clinical study to analyze the gut microbiota between colorectal cancer patients who developed AL and those who did not (nAL) using 16S-rRNA sequencing and quantitative real-time PCR to identify AL risk bacterial taxa. Then we built a rat anastomosis model and performed a bacteria transplantation to ensure the cause-effect relationship. The anastomotic healing score was used to evaluate the healing of anastomosis. In addition, we assessed the adhesion ability of bacteria by staining with fluorescein isothiocyanate and attachment assay. The expression of matrix metalloproteinase 9 (MMP9) was detected by western blot, and the activity was detected by gelatin zymography.

**Results:**

We found that the abundance and positive rate of *Fusobacterium nucleatum* (*Fn*) were higher in the AL patients. Exposure of the rat’s colon anastomosis to *Fn* contributes to the loss of submucosa collagen I and III, leading to AL’s pathogenesis. *Fn* can attach to the gut epithelial cells and stimulate intestinal MMP9 expression *in vitro* and *in vivo*. We further confirmed that these effects of *Fn* depended on the E-cadherin/β-catenin signaling pathway.

**Conclusion:**

This work demonstrates that *Fn* attaches and then stimulates the expression of epithelial cells MMP9 by the E-cadherin/β-catenin signaling pathway. These effects contribute to collagen break down in the intestinal tissue, finally leading to AL.

## Introduction

Anastomotic leak (AL) is a common and devastating complication following colorectal surgery, which causes high cancer recurrence rates and poor disease-free survival (Koedam et al., 2022). Despite advances in anastomotic techniques and perioperative care, the incidence of AL has not substantially decreased over time (Foppa et al., 2020). Several risk factors have been proven to be associated with AL, including male sex, obesity, smoking, and other intraoperative factors (McDermott et al., 2015), but the etiology and pathogenesis of AL remain unknown. There is accumulating evidence pointing out that AL is the result of a complex, dynamic interaction of several factors, especially the gut microbiota (GM) (Foppa et al., 2020).

In 1955, Cohn first demonstrated that antibiotic treatment improved anastomotic healing in dogs (Cohn and Rives, 1955). Since then, oral antibiotics have been introduced as a routine part of the preparation for gastrointestinal surgery. However, the antibiotic could disrupt the average defense ability of mucosa *via* depletion of the commensal GM, offering chances for pathogenic bacteria colonization (Buffie and Pamer, 2013). The lower strength of intestinal anastomoses in germ-free mice was seen, which suggests that normal microbiota may be beneficial and even necessary for optimal wound healing (Okada, 1994). It is reasonable that microbiota dysbiosis caused by surgery or prophylactic antibiotic therapy might worsen the patients’ gut microbiota, which is already unfavorable. In recent years, researchers have focused on discovering the mechanism of AL caused by pathogenic bacteria and finding a precise method to prevent AL (Shogan et al., 2015; Hyoju et al., 2018).

The structural protein collagen is essential in maintaining the intestinal wall’s strength. It was shown that high collagenase-producing strains, such as *Enterococcus faecalis* and *Pseudomonas aeruginosa*, could directly cause AL (Shogan et al., 2015; Jacobson et al., 2021). More than that, these pathogenic strains can also induce collagen breakdown by activating matrix metalloproteinase 9 (MMP9) in intestinal tissue contributing to the pathogenesis of AL (Shogan et al., 2015; van Praagh et al., 2020).

Although MMPs are a part of the physiologic reaction to tissue injury, however, supraphysiologic elevated levels cause a negative effect on stromal regeneration and potentially over-degradation of collagen structure (Lundy, 2014). *Fusobacterium nucleatum* (*Fn*) is a common oral gram-negative anaerobe that has gained significant attention for its potential tumorigenesis role in colorectal cancer (CRC). It is known that *Fn* can attach and invade colon epithelial cells through E-cadherin, leading to β-catenin activation and then promoting CRC (Rubinstein et al., 2013). However, *Fn* has not been reported to be related to AL.

Here, we found that the abundance of *Fn* was higher in AL patients, which drew our attention to know whether and how *Fn* contributed to the pathogenesis of AL. We have demonstrated that *Fn* existed in anastomotic tissues after surgery and stimulated MMP9 expression of the epithelial cell by activating the E-cadherin/β-catenin signaling pathway, finally leading to AL.

## Materials and methods

### Patients and clinical samples

The CRC patients who underwent colorectal resection from The First Affiliated Hospital of Harbin Medical University were included in this study. The exclusion criteria were as follows: patients younger than 18 years, emergent surgery, protective stoma, underlying severe diseases, and patients undergoing neoadjuvant chemoradiotherapy. AL was identified according to the “International Study Group of Rectal Cancer” definition (Rahbari et al., 2010). When the operating surgeon removed the colon sample, the CRC tissues were frozen immediately in liquid nitrogen. At the first stage of clinical research, we collected CRC tissue samples from 10 AL patients and 10 age-matched nAL patients (Cohort 1) for 16S rRNA gene sequencing. To further verify the difference in the anastomosis tissue bacteria between AL and nAL patients, we expanded the number of patients in the original cohort and applied the new cohort (16 AL CRC patients versus 29 nAl CRC patients, Cohort 2) for specific bacterial taxa 16S rRNA genes qPCR.

### Rat model

Adult male Wistar rats (250 to 300 g; Charles River. Beijing) were used for all experiments. To eliminate the interference of other bacteria in rat’s anastomosis healing, antibiotics treatment were given 1 week before surgery by adding ampicillin (0.5 mg/ml), neomycin (0.5 mg/ml), metronidazole (0.5 mg/ml), and vancomycin (0.25 mg/ml) in the sterile drinking water. Then rats were anesthetized through an intraperitoneal injection of ketamine (100 mg/kg) and xylazine (10 mg/kg). A midline abdominal incision was created, and 1 cm of the colon was resected at the peritoneal reflection. An anastomosis was performed using 8–12 simple interrupted varus sutures using a 6–0 proline suture. Integrity was tested by distending the distal colon with saline *via* enema using a gavage needle. All rats were volume resuscitated with 2 ml normal saline, then closed the abdomen in 2 layers with 2–0 silk sutures. On postoperative day (POD) 1, rats were assigned randomly to the following treatment groups: Phosphate bufered solution (PBS) rectal enema (3 ml, PBS group); *Fn* rectal enema (5 × 10^8^ CFU *Fn* resuspended in 3 ml PBS, *Fn* group); Heat-killed *Fn* rectal enema (5 × 10^8^ CFU heat-killed *Fn* resuspended in 3 ml PBS, H-K *Fn* group); *E.coli* rectal enema (5 × 10^8^ CFU *E.coli* resuspended in 3 ml PBS, *E.coli* group). In all groups, the rectal enema was performed twice daily using a 22G blunt tip gavage needle and continued until POD3. On POD7, rats were euthanized, and the anastomotic tissue samples were collected for microbial and histologic examination. For rescue experiments, genistein (10 mg/kg) was administered once daily on POD1-3 through intraperitoneal injection. The animal experiment was repeated twice.

The anastomotic healing score (AHS) was determined using the following scale: 0, normal healing; 1, flimsy adhesions; 2, dense adhesions without abscess or intraperitoneal contamination; 3, dense adhesions with a gross abscess at the anastomotic site; and 4, gross leak with peritoneal contamination and visible anastomotic dehiscence (Hyoju et al., 2018).

### Cell culture

The human epithelial cell line Caco-2 and FHC were purchased from the Shanghai Institute of Cell Biology (Shanghai, China) and maintained in DMEM or 1,640 with 10% foetal bovine serum (FBS) and 1% penicillin–streptomycin. The cells were cultured at 37°C in a humidified 5% CO2 atmosphere.

### Bacterial cultivation and heat-killed

*Fn* strain ATCC 25586 was purchased from American Type Culture Collection (ATCC, Manassas, VA) and cultured overnight at 37°C under anaerobic conditions in brain-heart infusion (BHI) supplemented with hemin, K2HPO4, vitamin k1, and l-cysteine. The commensal *Escherichia coli* strain DH5a (Tiangen, China) was cultured in luria-bertani (LB) medium overnight at 37°C in shake cultivation at 220 rpm/min. For heat-killed, the bacterial suspension was pelleted, washed with PBS, and then heat-inactivated at 100°C for 10 min in an autoclave. Subsequently, the heat-killed bacterial suspension was replated onto blood agar and cultured anaerobically for 48 h to confirm the death of the bacteria (Supplementary Figure S1).

### 16 s rRNA gene sequencing and bioinformatic analysis

16 s rRNA gene sequencing and bioinformatic analysis were followed as previously described (Jin et al., 2022). The human CRC samples were collected, and microbial DNA was extracted using TRIzol® Reagent according to the manufacturer’s instructions (Invitrogen). The V3-V4 hypervariable regions of the bacterial 16S rRNA gene were amplified. Purified amplicons were pooled in equimolar concentrations, and paired-end sequenced (2 × 300) on an Illumina MiSeq platform (Illumina, San Diego, United States) following standard protocols recommended by Majorbio Bio-Pharm Technology (Shanghai, China). Wilcoxon rank-sum test was used to detect features with significantly different abundance levels between assigned taxa based on a normalized relative abundance matrix. Profile clustering patterns from BrayCurtis distance measures were analysed using ANOSIM and betadisper tests from the vegan package. All tests were performed using 999 permutations.

### Gelatin zymography

To measure the activity of MMPs, conditioned medium and anastomotic tissues were prepared as follows. Epithelial cells were cultured on 6-well plates to reach 80% confluency, then co-cultured with *Fn* at an MOI of 300:1 for 24 h in the absence of serum. Used a 0.22 μm filter to remove bacteria and cells from the medium, then collected and stored the conditioned medium at −80°C. For anastomotic tissues, 50 mg of anastomotic tissues were lysed using RIPA lysis buffer with 1 mmol/l phenylmethyl sulfonyl fluoride (PMSF) for 30 min. After centrifugation, the supernatants were collected and dissolved in a sample buffer. Quantified amounts (30 μg in each well) of protein were separated in 10% SDS–polyacrylamide gels containing 1 mg/ml gelatin (Sigma-Aldrich, Germany). After electrophoresis, the gels were placed in a constant temperature shaker at 37°C, 50 r/min conditions and washed 4 times for 15 min each with washing buffer (50 mMTris-HCl [pH 7.5], 5 mM CaCl2, 1 μM ZnCl2, 2.5% Triton X-100) followed by 20 min wash with incubation buffer (washing buffer without Triton X-100) for 2 times. Then incubated the gels for 42 h in incubation buffer, stained with 0.5% coomassie blue R250 for 3 h, and destained with destaining solution (30% methanol, 10% acetic acid) until a clear band of degraded gelatin by MMPs was shown in the blue background gel.

### Cell attachment assay

Epithelial cells were seeded in a 6-well plate and grown to 100% confluency. Before infection, the cells were washed twice with PBS to remove antibiotics. Bacteria were added to the cells at an MOI of 100:1 and incubated at 37°C in a 5% CO2 incubator for 1 h. Then the monolayers were washed with PBS 3 times to remove the unattached bacteria. Subsequently, 1 ml of sterile distilled water was added to each well to lyse cells for 20 min. Serial dilutions of the lysates were plated onto blood agar plates to enumerate the attached bacteria. Attachment efficiency was expressed as the percentage of bacteria retrieved following cell lysis relative to the total number of bacteria initially added.

### H&E and Masson’s trichrome staining

The anastomotic tissues were washed, dehydrated, and embedded in paraffin after being fixed in 4% paraformaldehyde for 48 h. The specimens were sectioned at 4 μm thickness by a microtome. Before staining, paraffin sections were dewaxed with xylene, rehydrated in graded ethanol, and then stained the sections according to the instruction of the HE Staining Kit (Solarbio, Beijing, China) and Masson’s Trichrome Stain Kit (Solarbio, Beijing, China). After dehydrating with xylene, the slides were preserved with a neutral resin.

### Immunohistochemical staining

After dewaxed, rehydrated, and washed in PBS, the sections were treated with 3% hydrogen peroxide to abolish endogenous peroxidase activity. Antigen retrieval was performed in Tris-EDTA buffer (pH 9.0). Blocked the sections with 5% BSA for 1 h, then incubated with primary antibody [MMP9 (1,300), Collagen Type I (1:500), Collagen Type III (1,500)] overnight at 4°C. The slices were restored to room temperature the next day and incubated with a second antibody for 1 h at 37°C. After washing with PBS three times, the slides were incubated with 3,3′-diaminobenzidine (DAB) solution and counterstained with hematoxylin.

### Fluorescence *in situ* hybridization

A Cy3- conjugated *Fn* 16S rRNA probe (5′-CTT GTA GTT CCG C(C/T) TAC CTC-3′) was used for the FISH assay to detect the existence of *Fn* (Yu et al., 2016). In brief, the dewaxed and rehydrated paraffin tissue sections were treated with proteinase K and fixed with 1% paraformaldehyde, then incubated with the pro-hybridized buffer for 3 h at 37°C. A mixture of hybridization buffer and the probe was used to incubate the sections for 18 h in a dark chamber at 42°C. After counterstaining with DAPI, the images were captured with a fluorescence microscope.

### Double immunofluorescence labeling

Similar to immunohistochemistry, the sections were treated with 3% hydrogen peroxide and Tris-EDTA buffer (pH 9.0). Permeabilized the sections with 1% Triton X-100 and blocked with 5% BSA for 1 h, then incubated with primary antibody [MMP9 (1:100), E-cadherin (1,100)] overnight at 4°C. After incubating with fluorescent secondary antibody, the slides were counterstained with DAPI, the images were captured with a fluorescence microscope.

### Statistical analysis

All categorical data are presented as the number of cases and percentages, while continuous data are shown as either means ± standard deviation (SD) or median with range. Categorical data were compared by the Pearson Chi2 test and continuous data by the independent sample t-test or Mann–Whitney U test. The logistic regression model was used to determine the independent risk factors. Statistical analysis was performed using SPSS 23.0 and Prism 8.0 software. Differences were considered significant when *p < 0.05, **p < 0.01, ***p < 0.001, ****p < 0.0001.

## Results

### *Fn* is a risk factor for AL in CRC patients following surgery

**Figure 1:**
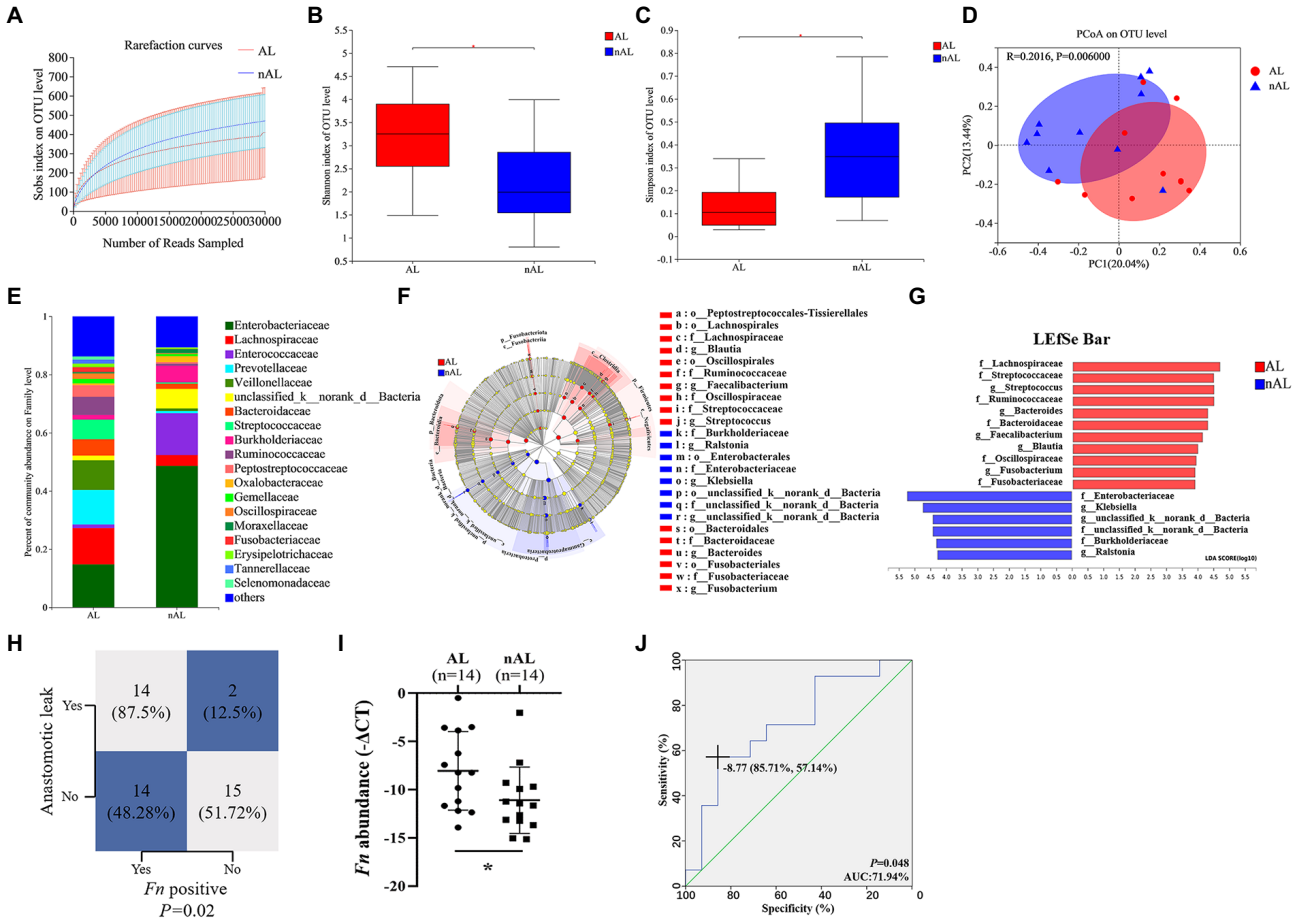
*Fn* is a risk factor for AL in CRC patients following surgery. **(A)** Rarefaction curves of species richness between the AL and nAL group. **(B,C)** Alpha diversity (Shannon index and Simpson index) between AL and nAL group. **(D)** The PCoA analyses based on BrayCurtis distance were used to reveal the β-diversity between AL and nAL group, ANOSIM statistical analysis. **(E)** The relative abundance of microbiota between AL and nAL group (family level). **(F)** A cladogram representation of data in CRC patients between AL and nAL group by 16S rDNA sequencing. **(G)** LDA coupled with the effect size measurements identifies the significant abundance of data in **(F)**. **(H)** Statistical analysis was conducted based on the positive of *Fn* and AL rate in Cohort 2, Chi-square test. **(I)** Statistical analysis of the amount of *Fn* in Cohort 2, nonparametric Mann–Whitney test. **(J)** ROC analysis was conducted based on the amount of *Fn* in CRC. *p < 0.05.

To examine the potential relationship between the GM and AL, we analyzed the GM using 16S rRNA sequencing in 10 CRC tissues from patients with AL and 10 matched control CRC tissues without AL (Cohort 1). The dilution curves of Alpha diversity indices were plotted to demonstrate adequate sequencing depth (Figure 1A). The Shannon and Simpson indexes of the AL group were significantly different from those of the nAL group on the OTU level (Figures 1B,C). The PCoA analyses based on BrayCurtis distance further revealed the significant difference in β-diversity between the two groups (Figure 1D). The bacterial community of CRC tissue samples was clustered according to whether patients occured AL (ANOSIM R = 0.202, *p* = 0.006, Supplementary Figure S2). The difference between each groups was more significant than that within the group, and grouping according to whether patients occured AL was meaningful. Betadisper analysis indicated that the significant ANOSIM result was not due to nonhomogeneous group dispersion (betadisper *F* = 0.988, *p* = 0.333, Supplementary Figure S3). The relative abundance of GM between the two groups on the family level was shown in Figure 1E. These results indicated that the microbial diversity and structure in the AL group was significantly different from that of the nAL group. We used the LEfSe algorithm to define the potential differential bacterium patterns between AL and nAL patients. We found that *Lachnospiraceae* and *Bacteroidaceae* were higher in the AL group, which is accorded with previous studies (Shogan et al., 2014; van Praagh et al., 2019). In addition, we also noticed that the *Fusobacteriaceae* family was also enriched in the AL group. On the genus level, the relative abundance of *Fusobacterium* (*p* = 0.008) in the AL group was significantly higher (Figures 1F,G). To confirm this result, we quantified the abundance of *Fn* by using qPCR in Cohort 2 and found that the *Fn* positive rate in the AL group was significantly higher than in the nAL group (87.5 vs. 48.28%, *p* = 0.02; Figure 1H). Among *Fn*-positive patients, we further confirmed that *Fn* abundance was significantly higher in the AL group (Figure 1I). Also, logistic regression analyses demonstrated that *Fn* was an independent risk factor for AL (OR 22.308, Table 1). Receiver operating characteristic (ROC) curve showed that the *Fn* abundance can well predict AL (AUC = 71.94%, *p* = 0.048; Figure 1J). These results suggested that *Fn* is a risk factor for AL.

**Table 1:** Univariate and multivariate analyses of risk factors for AL.

Factor category	Event rate %	Univariate analysis			Multivariate analysis		
		Odds ratio	95% CI	*p*	Odds ratio	95% CI	*p*
Fusobacterium nucleatum	Negative 11.8	1			1		
	Positive 50.0	7.5	1.439–39.089	0.017	22.308	1.491–333.775	0.024
Sex	Female 20.8	1			1		
	Male 52.4	4.180	1.133–15.419	0.032	4.898	0.693–34.622	0.111
Smoker	Nonsmoker 25.7	1			1		
	Smoker 70.0	6.741	1.430–31.773	0.016	10.202	1.142–91.169	0.038
BMI	<28 kg/m^2^ 28.2	1			1		
	≥28 kg/m^2^ 83.3	12.727	1.331–121.658	0.027	20.542	0.912–462.952	0.057
Tumor height from anal verge on rigid sigmoidoscopy	≤7 cm 71.4	1			1		
	>7 cm 28.9	0.163	0.027–0.970	0.046	0.100	0.008–1.210	0.070
Tumor size	≤5 cm 34.3	1					
	>5 cm 40.0	1.278	0.301–5.420	0.740			
Preoperative albumin	<34 g/dl 57.1	1					
	≥34 g/dl 31.6	0.487	0135–1.758	0.272			
Preoperative carcinoembryonic antigen	≤3.4 ng/ml 26.3	1					
	>3.4 ng/ml 42.3	2.053	0.569–7.413	0.272			
ASA score	I−II 28.6	1					
	III−IV 41.7	1.786	0.513–6.214	0.362			
Diabetic	Nondiabetic 37.5	1					
	Diabetic 20.0	0.417	0.042–4.085	0.452			
Age	<60 50.0	1					
	≥60 29.0	0.409	0.111–1.506	0.179			
Anemia	Nonanemia 31.3	1					
	anemia 46.2	1.886	0.503–7.073	0.374			

### *Fn* degrade collagen by stimulating the MMP9 expression of epithelial cells and contribute to AL

**Figure 2:**
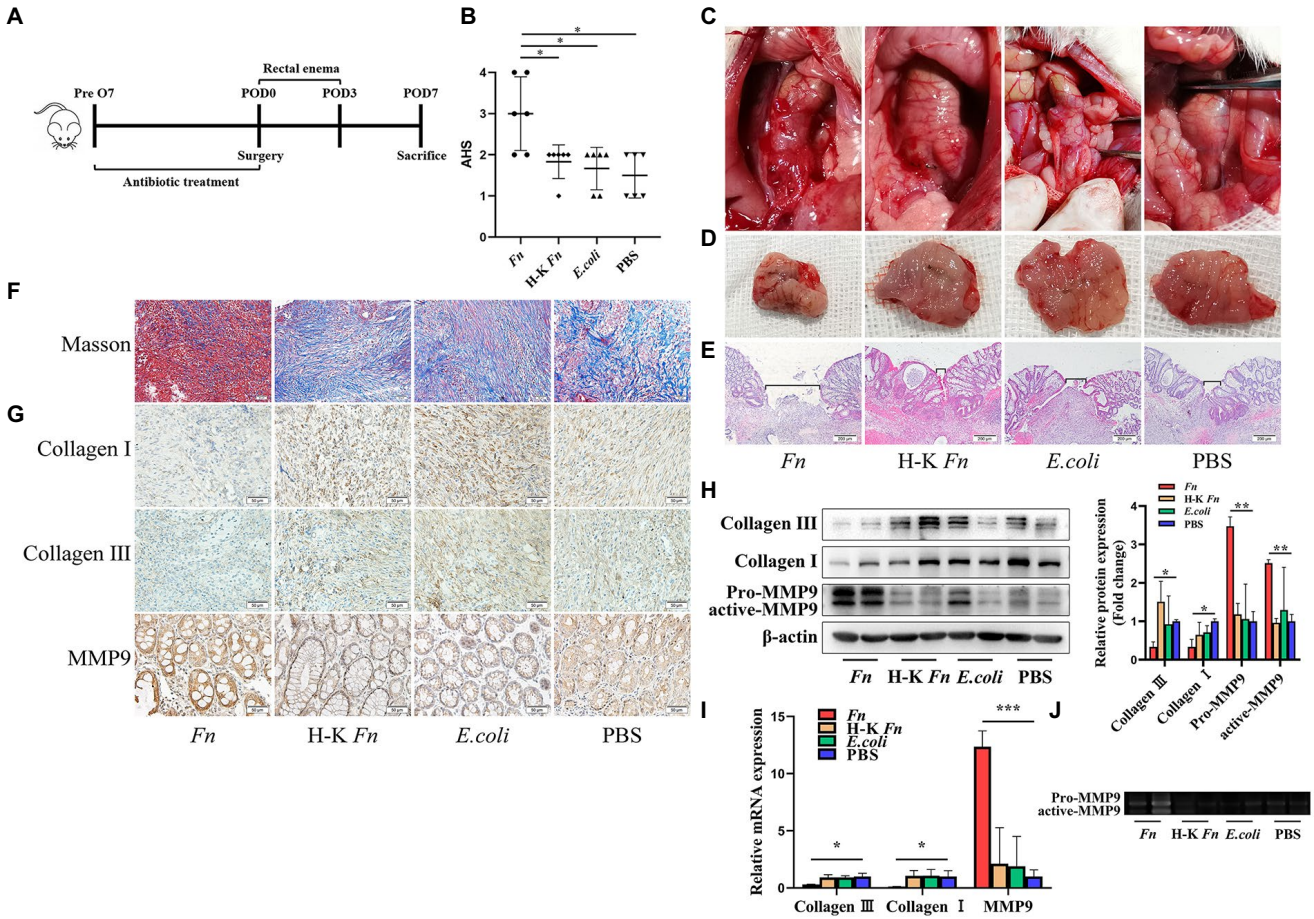
*Fn* degrade collagen by stimulating the MMP9 expression of epithelial cells and contribute to AL. **(A)** Sketch of anastomosis model. Rats were assigned randomly to the following treatment groups: PBS rectal enema (PBS group); *Fn* rectal enema (*Fn* group); Heat-killed *Fn* rectal enema (H-K *Fn* group); *E.coli* rectal enema (*E.coli* group). **(B)** The AHS of four groups (n = 6 per group). **(C)** Gross inspection of the anastomotic healing. Only *Fn* group rats have anastomotic dehiscence. **(D)** Excised and exposed suture lines of anastomotic sites. All suture lines are grossly intact except for the *Fn* group. **(E)** H&E staining of anastomotic tissues. Brackets indicate the width of tissue apposition at the suture line (original magnification, 40×) (bar, 200 μm). **(F)** Masson staining of anastomotic tissues demonstrates collagen depletion (original magnification, 200×) (bar, 50 μm). **(G)** Immunohistochemical staining shows both the expression of collagen, MMP9 and the location of MMP9 (original magnification, 200×) (bar, 50 μm). **(H)** The protein expression level of collagen and MMP9 in anastomotic tissues. **(I)** The RNA expression level of collagen and MMP9 in anastomotic tissues. **(J)** The activity of *Fn*-activated MMP9 was detected by gelatin zymography. *p < 0.05, **p < 0.01, ***p < 0.001.

As a member of *Fusobacterium*, *Fn* is a highly abundant species in CRC patients and plays a significant role in carcinogenesis. However, the potential relationship between *Fn* and AL remains unclear. To identify the role of *Fn* on anastomosis healing, we built up an AL model in rats by performing a distal colon resection and anastomosis followed by intestinal inoculation with *Fn*, H-K *Fn*, *E.coli*, and PBS *via* enema from POD1 to POD3. All rats were sacrificed on POD7 and underwent laparotomy for gross inspection of the anastomotic healing (Figure 2A). No leaks were observed in the control groups. In contrast, four of six rats in the *Fn* group developed a clinical leak (Figure 2B). We found that only rats in group *Fn* demonstrated evidence of a significant incidence of spontaneous AL with visible anastomotic dehiscence (Figure 2C), grossly visible disruption of the anastomotic suture line (Figure 2D), and poor tissue healing at suture line (Figure 2E).

Collagen in the submucosa can improve the toughness of anastomosis and directly affect the anastomosis healing (Thornton and Barbul, 1997). Histologic examination demonstrated visible collagen depletion in the *Fn* group (Figure 2F). Similarly, immunohistochemical staining showed a decrease in the number of Collagen I and Collagen III positive cells in the *Fn* group (Figure 2G). This result was confirmed by WB (Figure 2H) and qPCR (Figure 2I). Then we further explored the mechanism of collagen decomposition. We found that the expression of MMP9 was up-regulated in the *Fn* group (Figures 2H,I). We also tested other MMPs which take collagen I and collagen III as substrates and found no difference between the four groups (Supplementary Figure S4). Gelatin zymography analysis of tissue extracts further demonstrated higher MMP9 activity in the *Fn* group (Figure 2J). These results confirmed that *Fn* could degrade collagen by stimulating the expression of intestinal MMP9 and contribute to AL.

### The adhesion ability of *Fn* might be a prerequisite for its pathogenic role in AL

**Figure 3:**
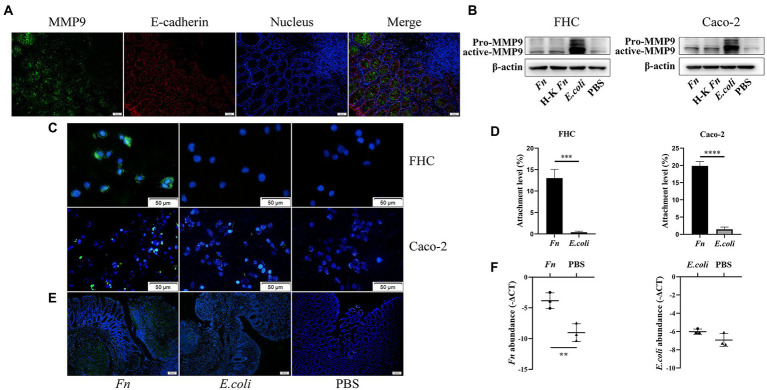
The adhesion ability of *Fn* might be a prerequisite for its pathogenic role in AL. **(A)** Double immunofluorescence labeling was used to observe MMP9 (green) and E-cadherin (red) location in anastomotic tissues (original magnification, 200×) (bar, 50 μm). **(B)** The protein expression level of MMP9 in epithelial cells treated with *Fn*, H-K *Fn*, *E.coli*, and PBS, respectively. **(C)** After washing with PBS, the amount of attached FITC-labeled *Fn* and *E.coli* (original magnification, 200×) (bar, 50 μm). **(D)** The ability of *Fn* and *E.coli* attached to epithelial cells was detected by cell attachment assay. **(E)** Rats were processed an enema with FITC-labeled *Fn* or *E.coli* on the POD1 and sacrificed 24 h later to measure their colonization ability (original magnification, 100×) (bar, 100 μm). **(F)** Relative abundance of *Fn* and *E.coli* in anastomotic tissues were calculated by qPCR. **p < 0.01, ***p < 0.001, ****p < 0.0001.

Consistent with a previous study (Castaneda et al., 2005), we confirmed the presence of MMP9 mainly in epithelial cells (Figure 2G). To further verify the source of MMP9, we applied double immunofluorescence labeling. We found MMP9 and epithelial marker E-cadherin exist in the same location (Figure 3A). It has been shown that *Fn* can stimulate MMP9 expression of oral epithelial cells (Mahtout et al., 2011). Thus, we hypothesized that *Fn* could attach and activate the gut epithelial cell to express MMP9. We co-cultured epithelial cells (Caco-2 and FHC) with *Fn*, heat-killed *Fn*, and *E.coli*, respectively. The results showed that *Fn* induced the expression of MMP9 in epithelial cells. To our surprise, the MMP9 expression of epithelial cells was also induced by *E.coli in vitro*, which is inconsistent with the *in vivo* results (Figure 3B).

Attachment and invasion are hallmarks of *Fn*. Thus, we hypothesized that the adhesion ability of *Fn* is the precondition for it to cause AL. We stained *Fn* and *E.coli* with fluorescein isothiocyanate (FITC) and then co-cultured with epithelial cells to assess their adhesion ability. We found that *Fn* adhesive ability was more potent than *E.coli* (Figure 3C). We further processed attachment assays *in vitro*. We confirmed that *Fn* had a stronger adhesion ability than *E.coli in vitro* (Figure 3D; Supplementary Figure S5).

*In vivo*, to confirm the colonization ability of *Fn* and *E.coli*, rats were processed an enema with FITC-labeled *Fn* or *E.coli* on the POD1 and sacrificed 24 h later to measure their colonization ability. We found that there are more *Fn* colonized on the anastomosis site than *E.coli* (Figure 3E). qPCR results further confirmed our hypothesis (Figure 3F). These findings demonstrate that the adhesion ability of *Fn* is necessary for its pathogenic role in AL.

### *Fn* promotes MMP9 expression of epithelial cells depending on the E-cadherin/β-catenin signaling

**Figure 4:**
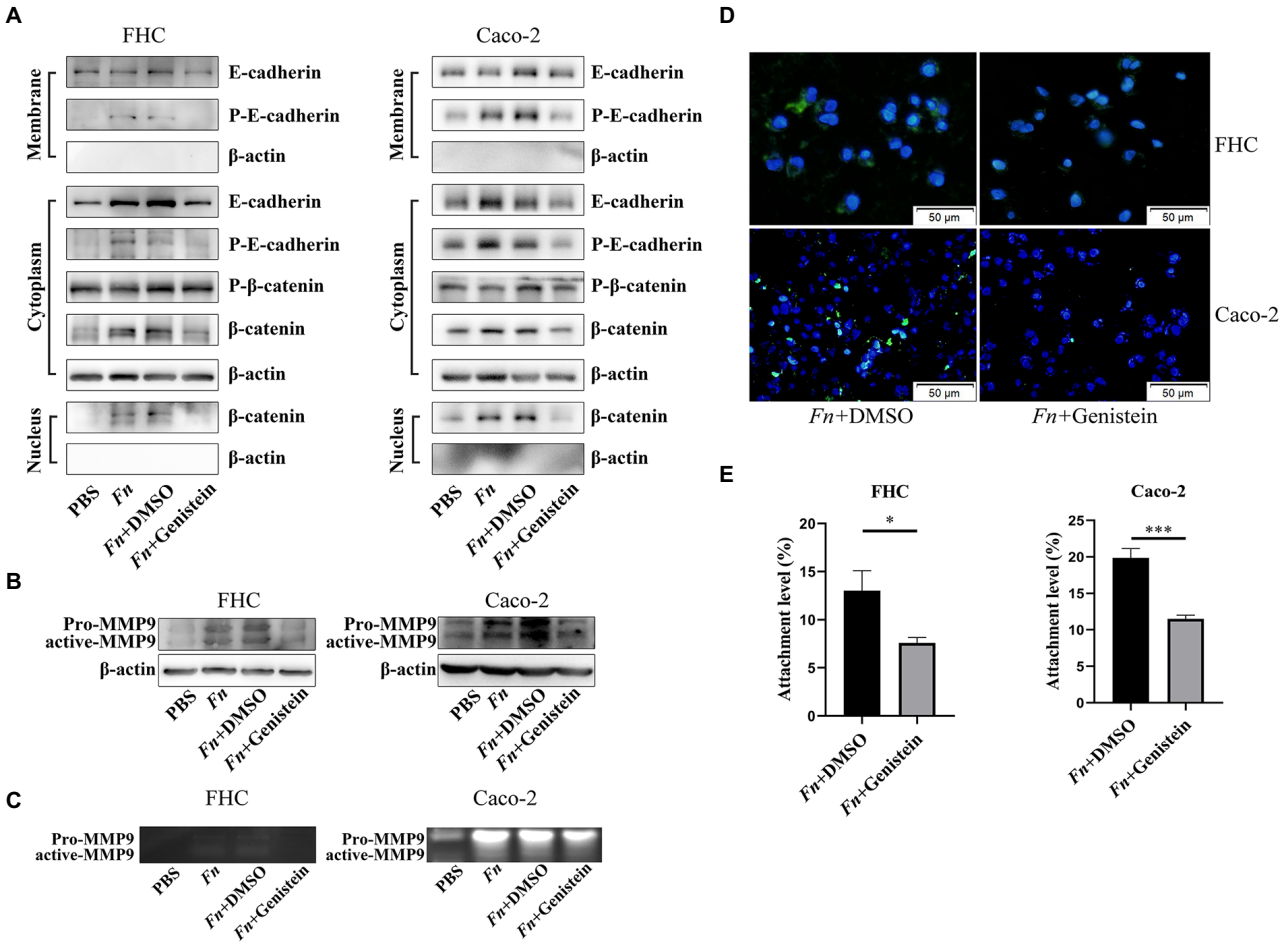
*Fn* promotes MMP9 expression of epithelial cells depending on the E-cadherin/β-catenin signaling. **(A,B)** The protein expression level of E-cadherin, β-catenin, and MMP9 in epithelial cells treated with PBS, *Fn*, *Fn* + genistein, and *Fn* + DMSO, respectively. **(C)** Gelatin zymography of four groups conditioned medium. **(D)** The amount of FITC-labeled *Fn* after genistein treatment (original magnification, 200×) (bar, 50 μm). **(E)** Genistein inhibits *Fn* from attaching to epithelial cells. *p < 0.05, ***p < 0.001.

We next addressed the mechanism by which *Fn* stimulated epithelial cell MMP9 expression. We have identified adhesion ability of *Fn* is significant for its pathogenic role. It has been reported that FadA, an adhesin of *Fn*, promotes its invasion and carcinogenesis effects on colon epithelial cells by modulating E-cadherin/β-catenin signaling (Rubinstein et al., 2013). Given that MMP9 are direct target genes of the Wnt signaling pathway (Wu et al., 2007), we hypothesized that *Fn* stimulates intestinal MMP9 expression by E-cadherin/β-catenin signaling. *In vitro*, we confirmed that *Fn* induced membrane E-cadherin phosphorylation and then internalization. This was accompanied by β-catenin accumulation in the cytoplasm and translocation into the nucleus resulting in the expression and activation of MMP9 (Figures 4A–C).

It was previously shown that protein tyrosine kinase (PTK) plays a crucial role in E-cadherin endocytosis and recycling (Le et al., 2002). We preincubated cells with 50 mM of genistein for 1 h and found that the PTK inhibitor genistein prevented E-cadherin phosphorylation and internalization, which decreased β-catenin translocation into the nucleus, and finally attenuated *Fn*-induced MMP9 expression (Figures 4A–C). Interestingly, genistein also prevented *Fn* attachment of epithelial cells (Figures 4D,E; Supplementary Figure S6). These results indicated that *Fn* attachment was required to activate E-cadherin/β-catenin signaling and then promote MMP9 expression.

### *Fn* promotes MMP9 expression and then leads to AL depending on the E-cadherin/β-catenin signaling

**Figure 5:**
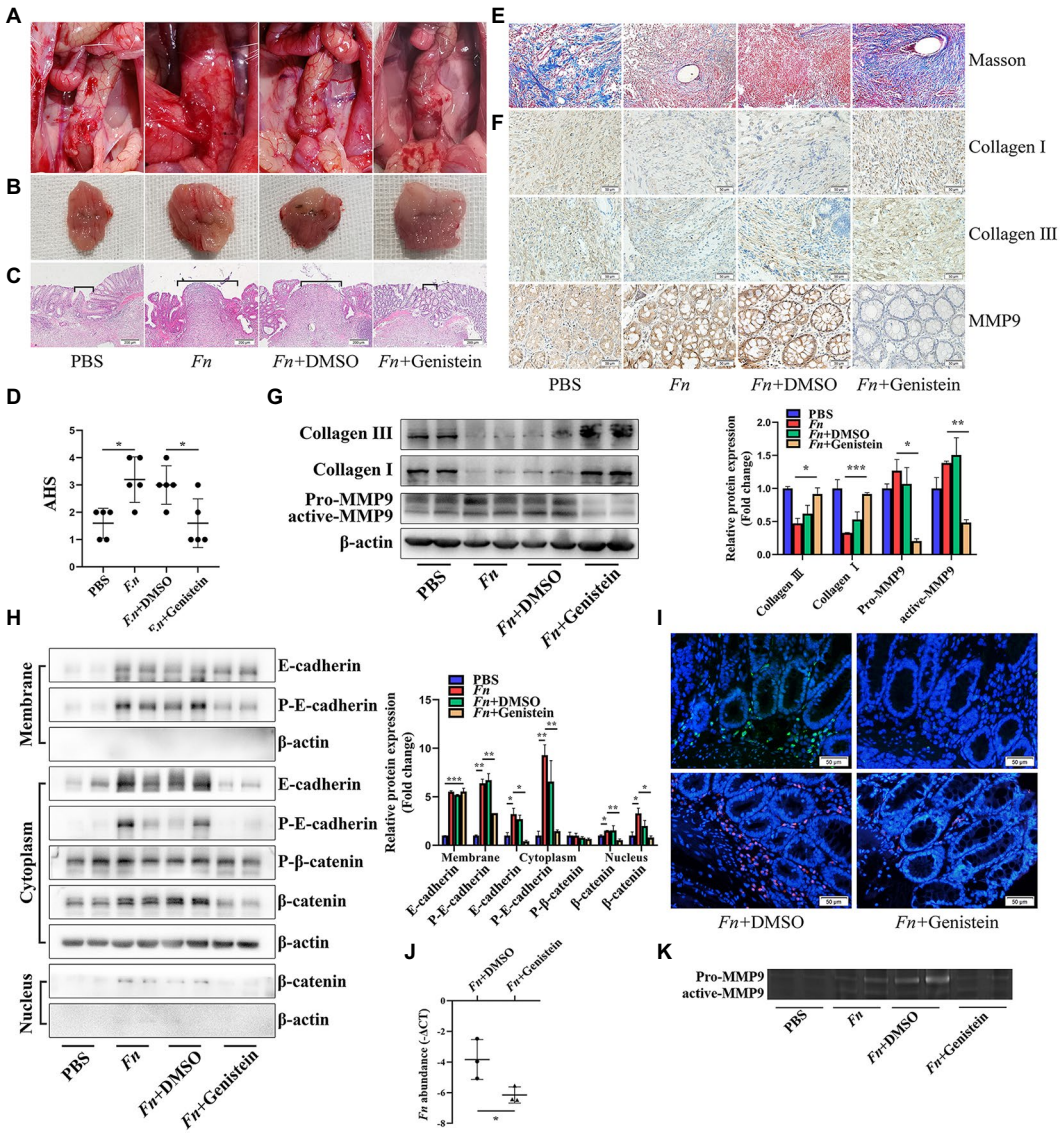
*Fn* promotes MMP9 expression and then leads to AL depending on the E-cadherin/β-catenin signaling. **(A)** Gross inspection of the anastomotic healing. Genistein improves the *Fn*-mediated AL. **(B)** Excised and exposed suture lines of anastomotic sites. **(C)** H&E staining of anastomotic tissues. Brackets indicate the width of tissue apposition at the suture line (original magnification, 40×) (bar, 200 μm). **(D)** The AHS of four groups (n = 5 per group). **(E)** Masson staining of anastomotic tissues demonstrates collagen depletion (original magnification, 200×) (bar, 50 μm). **(F)** Immunohistochemical staining shows the expression of collagen and MMP9 (original magnification, 200×) (bar, 50 μm). **(G,H)** The protein expression level of E-cadherin, β-catenin, collagen, and MMP9 in anastomotic tissues. *Fn* activates the E-cadherin/β-catenin signaling pathway, stimulates the expression of MMP9, and contributes to collagen depletion. Genistein inhibits all *Fn*-activated functions. **(I)** FITC-labeled *Fn* (green) and Cy3-conjugated *Fn* (red) were used to detect the colonization ability (original magnification, 200×) (bar, 50 μm). **(J)** qPCR demonstrates that genistein prevents *Fn* from adhering to the anastomotic sites. **(K)** Gelatin zymography demonstrates that genistein inhibits the *Fn*-activated MMP9 in anastomotic tissue. *p < 0.05, **p < 0.01, ***p < 0.001.

To further explore if *Fn* stimulates the expression of intestinal MMP9 and contributes to AL depending on the E-cadherin/β-catenin signaling *in vivo*, we used the PTK inhibitor genistein to block the E-cadherin/β-catenin pathway in rats. As was expected, The *Fn*-mediated AL was abolished by genistein treatment. No leaks were observed in rats subjected to genistein (Figure 5D). Specifically, genistein alleviated bowel adhesions and improved anastomosis healing on the suture site (Figures 5A–C).

Histologic examination and WB demonstrated that the *Fn*-induced collagen depletion was also abolished by genistein treatment, which was confirmed by an immunohistochemical experiment (Figures 5E–G). Consistent with *in vitro* experiments, genistein not only inhibited E-cadherin phosphorylation and internalization (Figure 5H) but also prevented *Fn* from adhering to the anastomotic sites (Figures 5I,J), resulting in decreased MMP9 expression and activation (Figures 5G,K). All these results indicated that *Fn* adheres to and stimulates the expression of epithelial MMP9, then contribute to AL depending on the E-cadherin/β-catenin signaling pathway.

## Discussion

During daily clinical practice, if we accept the fact that some AL is inevitable, then an appropriate level of suspicion for investigating and identifying AL before our patients’ condition must begin to deteriorate. To our disappointment, many of the findings associated with AL are neither sensitive nor specific (Vallance et al., 2017). GM dysbiosis has been recognized to contribute to AL in the past decade, but the exact effects are still vague. Data from the present study demonstrate that the high abundance of *Fn* in CRC patients acts as a pathogen for AL. We have found that the positive rate of *Fn* on anastomosis tissue is higher in AL patients compared to the average rate of 48% in CRC patients in our study and others (Brennan and Garrett, 2019; Hashemi Goradel et al., 2019). This result can be explained by the fact that the routine practices of oral antibiotics in combination with MBP (magnesium sulfate solution) can not eliminate *Fn* from the gut, and the abundance is even higher in AL patients. In addition, we showed that the adhesion ability of *Fn* is necessary to induce the epithelial cell to express MMP9, which is the ultimate cause of poor anastomosis healing.

A suitable animal model would be the most desirable precondition for AL study. By now, there is no perfect animal model accepted by all. Most studies applied the rat model by colon resection and anastomosis build-up (Shogan et al., 2015; Hyoju et al., 2018). For bacterial transplantation, we adopt enema procedure similar to other studies (Olivas et al., 2012; Shogan et al., 2015). By inserting a 22G blunt tip gavage needle about 5−7 cm from the anus, we can reach the anastomotic site and accurately transplant bacteria. Referring to others studies on gut microbiota and AL, we suspect that enema would be better than oral transplantation for bacteria colonization on colon, especially on the anastomosis site where close to the anus. We confirmed intestinal inoculation with *Fn*, but not for heat-killed *Fn*, by anal enema deleterious anastomosis healing. This result showed that *Fn*’s viable character is necessary for its pathogenic role. A previous study also showed that heat-killed *Fn* could not activate the JNK pathway of gingival epithelial cells compared to viable *Fn*. This phenomenon is due to heat-killed *Fn* cannot invade gingival epithelial cells (Ji et al., 2009).

Another study shows that heat-killed *Fn* could upregulate the TLR-4 pathway in oral keratinocytes (Shao et al., 2021). TLR-4 is known to be responsible for recognizing LPS and activating this pathway results in increased production of inflammatory cytokines. This result indicated that without invasion ability, the LPS from heat-killed *Fn* can still activate the TLR-4. We found that *E.coli* can induce MMP9 expression (one of the upstream regulators is the TLR-4 pathway) of epithelial cells *in vitro* but not *in vivo*. All these results further confirmed that the adhesive ability of viable *Fn* is necessary for *Fn* to induce MMP9 expression *in vivo*.

It is known that the submucosa is significant for anastomotic healing. Indeed, this layer is the source of collagen, which can improve the toughness of anastomosis and directly affect the healing of anastomosis (Thornton and Barbul, 1997). Type I collagen (68%) and type III collagen (20%) are the most predominant type in submucosa (Thornton and Barbul, 1997), also studies have confirmed that type I collagen and type III collagen activated at the early stage of anastomotic healing (Braskén, 1991). The current study found that *Fn* inoculation resulted in Collagen I and III depletion on the submucosa.

Collagenase activity plays an essential role in the healing of anastomosis. Several pathogens expressing this collagenolytic phenotype, including *Pseudomonas aeruginosa*, *Serratia marcescens*, and *Enterococcus faecalis*, have been associated with leak development (Shogan et al., 2015; Hyoju et al., 2018). A previous study reported that the collagenolytic phenotype of *Enterococcus faecalis*, along with its capacity to activate MMP9 in the host’s intestinal tissue, leads to AL (Shogan et al., 2015).

Although MMPs are a part of the normal collagenolytic response to injury, elevated levels of MMPs negatively affect the process of stromal regeneration and potentially result in over-degradation of provisional collagen, then tissue breakdown (Shogan et al., 2015).

Nevertheless, *Fn* has not been reported as a collagenase-producing bacteria (Gendron et al., 2004). For this aspect, we hypothesized that *Fn* could degrade collagen by stimulating the expression of intestinal MMPs but not produce active MMPs *per se*. We found that *Fn* induced MMP9 instead of other MMPs activity in the anastomotic tissue of rats. To identify the source of overexpressed MMP9, we performed immunohistochemical staining and double immunofluorescence staining. We found that the epithelial cell is one of the MMP9 expression cells. It has been shown that *Fn* can modulate the expression and secretion of MMP9 by oral epithelial cells (Mahtout et al., 2011; Inaba et al., 2014). We co-cultured *Fn* with intestinal epithelial cells (Caco-2 and FHC) and found increased MMP9 expression. One unanticipated finding was that *E.coli* also showed MMP9 inducing effect, which was not seen in rats. A previous study has found that *E.coli* can induce MMP9 expression by the TLR-4/NF-kB pathway (Zhang et al., 2013). This inconsistency may be due to the vast difference between *in vitro* and *in vivo* studies. However, to our suspicion, *E.coli* losing this effect in the rat gut may result from that it can not persist on the anastomosis site after surgery. Regular gut transit helps to clean the gut luminal bacteria. That is to say, species with weak adhesive ability would be swept away (Roager et al., 2016).

On the contrary, *Fn* is equipped with the adhesive ability to epithelial cell and belong to the intracellular infection species (Rubinstein et al., 2013). As expected, we first found that *Fn* adhered to epithelial cells but not *E.coli in vitro*. Then, we conducted an *in vivo* study by performing bacteria solution enema and confirmed that there are more *Fn* colonized on the anastomosis site than *E.coli*. So, we can conclude that the adhesive ability of *Fn* is necessary for its pathogenic role in AL.

A group of proteins exposed on the pathogen’s surface called “adhesins” has been identified as the molecular basis for *Fn* adherence to specific host molecules (Brennan and Garrett, 2019). Even though we did not know which adhesins of *Fn* should be blamed in this study. However, the FadA, one of the essential adhesins on *Fn*, has been reported to interact with E-cadherin of the epithelial cell to realize the adhesive effect of *Fn*, then modulating E-cadherin/β-catenin signaling in CRC (Rubinstein et al., 2013). We found that *Fn* induced membrane E-cadherin phosphorylation, then internalization, accompanied by β-catenin accumulation in the cytoplasm, then translocation into the nucleus resulting in expression and activation of MMP9. Inhibiting E-cadherin internalization by genistein block not only these effects of *Fn* but also intercept *Fn* attaching to epithelial cell. We suspect that preventing *Fn* from attaching on epithelial might be a potential therapeutic way to improve anastomosis healing. In fact, we found that genistein block the E-cadherin/β-catenin pathway and *Fn* attaching effect *in vivo*, then prevent AL.

There are some limitations to our study. Our results from patients undergoing colorectal surgery were not powered to determine the positive predictive value of *Fn* on AL. However, our preclinical data suggest that it may be worth testing the predictive value of *Fn* on AL. Owing to the complex biological processes involved, it is challenging to mimic anastomotic healing in vitro. A recent systematic review showed that the study of anastomotic failure in animals exists several disadvantages (Yauw et al., 2015). Even though several classical studies still apply the rat model as we did to interpret the role of GM in AL. There are technical challenges in creating an intestinal wound and then directly observing anastomosis healing in an internal organ in a clinically relevant manner. Applying organoids to investigate the molecular process of intestinal healing in more detail would be ideal (Giesen et al., 2014).

In summary, we present evidence that *Fn* attaches to gut epithelial cells and subsequently stimulates MMP9 expression, which breaks down collagen that underlies AL. This study suggests that blocking *Fn* attachment to epithelial cells is a potential strategy to prevent AL.

## Data availability statement

The original contributions presented in the study are included in the article/Supplementary material, further inquiries can be directed to the corresponding authors. The 16S rRNA Amplicon Sequencing data was deposited in Sequence Read Archive (SRA) database: PRJNA705612 and PRJNA704516.

## Ethics statement

The animal study was performed by The First Affiliated Hospital of Harbin Medical University protocol (201909). The clinical trial was approved by the Research Ethics Committee of the First Affiliated Hospital of Harbin Medical University (IRB-AF/SC-08/05.0). All participants in this study provided signed informed consent.

## Author contributions

SS, YL, and ZW conceived the study design and performed the experiments. XJ, WY, and recruited and XG followed up the patients. BLin, HW, and BLi performed the sequencing analysis and contributed to the data analyses. SS, YL, JZ, and YW drafted the manuscript. All authors contributed to the article and approved the submitted version.

## Funding

This work was supported by the National Natural Science Foundation of China (81970466) and Ningbo Clinical Research Center for Digestive System Tumors (2019A21003).

## Conflict of interest

The authors declare that the research was conducted in the absence of any commercial or financial relationships that could be construed as a potential conflict of interest.

## Publisher’s note

All claims expressed in this article are solely those of the authors and do not necessarily represent those of their affiliated organizations, or those of the publisher, the editors and the reviewers. Any product that may be evaluated in this article, or claim that may be made by its manufacturer, is not guaranteed or endorsed by the publisher.

## Figures and Tables

**Table:** 

AL	Anastomotic leak
MMP9	Matrix metalloproteinase 9
Fn	Fusobacterium nucleatum
GM	Gut microbiota
CRC	Colorectal cancer
POD	Postoperative day
AHS	Anastomotic healing score
FBS	Foetal bovine serum
BHI	Brain-heart infusion
LB	Luria-Bertani
PMSF	Phenylmethyl sulfonyl fluoride
DAB	3,3′-diaminobenzidine
FISH	Fluorescence *in situ* hybridization
SD	Standard deviation
ROC	Receiver operating characteristic
FITC	Fluorescein isothiocyanate
PTK	Protein tyrosine kinase
PBS	Phosphate bufered solution
PBST	Phosphate-buffered saline with Tween 20
qPCR	Quantitative real-time PCR
