# Supplementary material for: Fusobacterium nucleatum induces colon anastomosis leak by activating epithelial cells to express MMP9
Source: Front Microbiol. 2022 Dec 14;13:1031882. doi: 10.3389/fmicb.2022.1031882 (PMC9794562; doi:10.3389/fmicb.2022.1031882)
Supplement: Supplementary file 1 [file Table_1.docx]

**Supplemental Material**

**Method**

**DNA extraction and bacterial DNA quantification by qPCR**

Bacterial DNA from human CRC samples and rat anastomotic samples was extracted following the instruction of the TIANamp Genomic DNA Kit (DP304, Tiangen, Beijing, China). Amplification and detection of *Fn* were performed with THUNDERBIRD SYBR® qPCR Mix (QPS-201. Toyobo Co. Ltd) using the CFX96 Touch System (BioRad). Reaction conditions were as follows: 1 min at 95°C, and 40 cycles of 15 sec at 95°C, 30 sec at 55°C, and 1 min at 72°C. Relative abundance was calculated by the -ΔCT method. Universal Eubacteria 16S was used as an internal reference for rat samples, and PGT was used for human samples. The primers used are listed in Table S1.

**RNA extraction and qPCR**

RNA extraction and qPCR were performed as previously described ^[1]^. The primers used are listed in Table S1. Relative abundance was calculated by the 2^-ΔΔCt^ method. qPCR was done in triplicates, and the expression levels of target genes were normalized to GAPDH.

**Western blot**

The total protein of *Fn* co-cultured cells or anastomotic tissues was extracted with RIPA lysis buffer (Solarbio, Beijing, China) mixed with PMSF. After vortexing and 30 minutes of incubation on ice, the lysates were centrifuged at 10,000 rpm for 10 minutes at 4°C. Collected the supernatants and used ND-2000 (NanoDrop Technologies) to quantify the concentration of total protein, then boiled the supernatants for 10 minutes with sample buffer. Proteins in the cell membrane, cytoplasm, and nuclei were extracted using the Membrane and Cytosol Protein Extraction Kit (Beyotime, Jiangsu, China) and Nuclear Protein Extraction Kit (Solarbio, Beijing, China).

An equal amount of protein was separated in 10% SDS-PAGE gels and transferred to PVDF membranes. The membranes were blocked using 5% nonfat dry milk in phosphate-buffered saline with Tween 20 (PBST) for 1.5 hours and then incubated with primary anti-MMP-9 (1:500; 10375-2-AP, Proteintech, China), Collagen Type I (1:1000; 14695-1-AP, Proteintech, China), Collagen Type III (1:1000; 22734-1-AP, Proteintech, China), phosphorylated E-cadherin (1:1000; ab76319, Abcam, UK), E-cadherin (1:1000; 20874-1-AP, Proteintech, China), phosphorylated β-Catenin (1:1000; WL03554, Wanleibio, China), β-Catenin (1:1000; WL0962a, Wanleibio, China), and β-Actin (1:1000; A01010, Abbkine, China) antibodies overnight at 4℃. Subsequently, the membranes were washed with PBST and incubated with HRP-conjugated secondary antibodies (goat anti-mouse or goat anti-rabbit IgG) (1:4000; A01010, Abbkine, China) for 1 hour at room temperature. After being exposed to SuperSignal West Pico Chemiluminescent Substrate (K22020, Abbkine, Beijing, China), the membranes were detected and quantified by using ImageLab software (Bio-Rad, Hercules, CA, USA).

Table S1 Primers of RT-qPCR

| Name | Sequence 5’-3’ |
| --- | --- |
| *E.coli*-F | CATGCCGCGTGTATGAAGAA |
| *E.coli*-R | CGGGTAACGTCAATGAGCAAA ^[2]^ |
| Fusobacterium spp-F | CGGGTGAGTAACG CGTAAAG |
| Fusobacterium spp-R | ACATTGTGCCACG GACATCTTG ^[3]^ |
| PGT-F | ATCCCCAAAGCACCTGGTTT |
| PGT-R | AGAGGCCAAGATAGTCCTGGTAA ^[4]^ |
| 16S-F | GTGSTGCAYGGYTGTCGTCA |
| 16S-R | ACGTCRTCCMCACCTTCCC ^[2]^ |
| Rat Collagen I-F | CAATGCTGCCCTTTCTGCTCCTTT |
| Rat Collagen I-R | ATTGCCTTTGATTGCTGGGCAGAC ^[1]^ |
| Rat Collagen III-F | ACTGGTGAACGTGGCTCTAA |
| Rat Collagen III-R | GGACCTGGATGTCCACTTGA ^[5]^ |
| Rat MMP9-F | AGGTGCCTCGGATGGTTATCG |
| Rat MMP9 -R | TGCTTGCCCAGGAAGACGAA ^[6]^ |
| Rat GAPDH-F | GAGCGTGGCTATTCCTTCGTG |
| Rat GAPDH-R | CAGTGGCCATCTCATTTTCAAAGT ^[1]^ |

Table S2 baseline characteristics of all patients

| **Factor category** | **N= 45** |
| --- | --- |
| Sex |  |
| Male | 21(46.7) |
| Female | 24(53.3) |
| Age |  |
| ＜60 | 14(31.1) |
| ≥60 | 31(68.9) |
| ASA score |  |
| Ⅰ-Ⅱ | 21(46.7) |
| Ⅲ-Ⅳ | 24(53.3) |
| BMI |  |
| ＜28 kg/m^2^ | 39(86.7) |
| ≥28 kg/m^2^ | 6(13.3) |
| Smoker |  |
| Nonsmoker | 35(77.8) |
| Smoker | 10(22.2) |
| Diabetic |  |
| Nondiabetic | 40(88.9) |
| Diabetic | 5(11.1) |
| Tumor height from anal verge on rigid sigmoidoscopy |  |
| ≤7 cm | 7(15.6) |
| ＞7 cm | 38(84.4) |
| Tumor size, (cm) |  |
| ≤5 cm | 35(77.8) |
| ＞5 cm | 10(22.2) |
| Preoperative albumin |  |
| ＜34 g/dL | 7(15.6) |
| ≥34 g/dL | 38(84.4) |
| Preoperative carcinoembryonic antigen |  |
| ≤3.4 ng/mL | 19(42.2) |
| ＞3.4 ng/mL | 26(57.8) |
| Anemia |  |
| Nonanemia | 32(71.1) |
| anemia | 13(28.9) |
| Fusobacterium nucleatum |  |
| Negative | 17(37.8) |
| Positive | 28(62.2) |
| Data are expressed as number of patients (%) | |


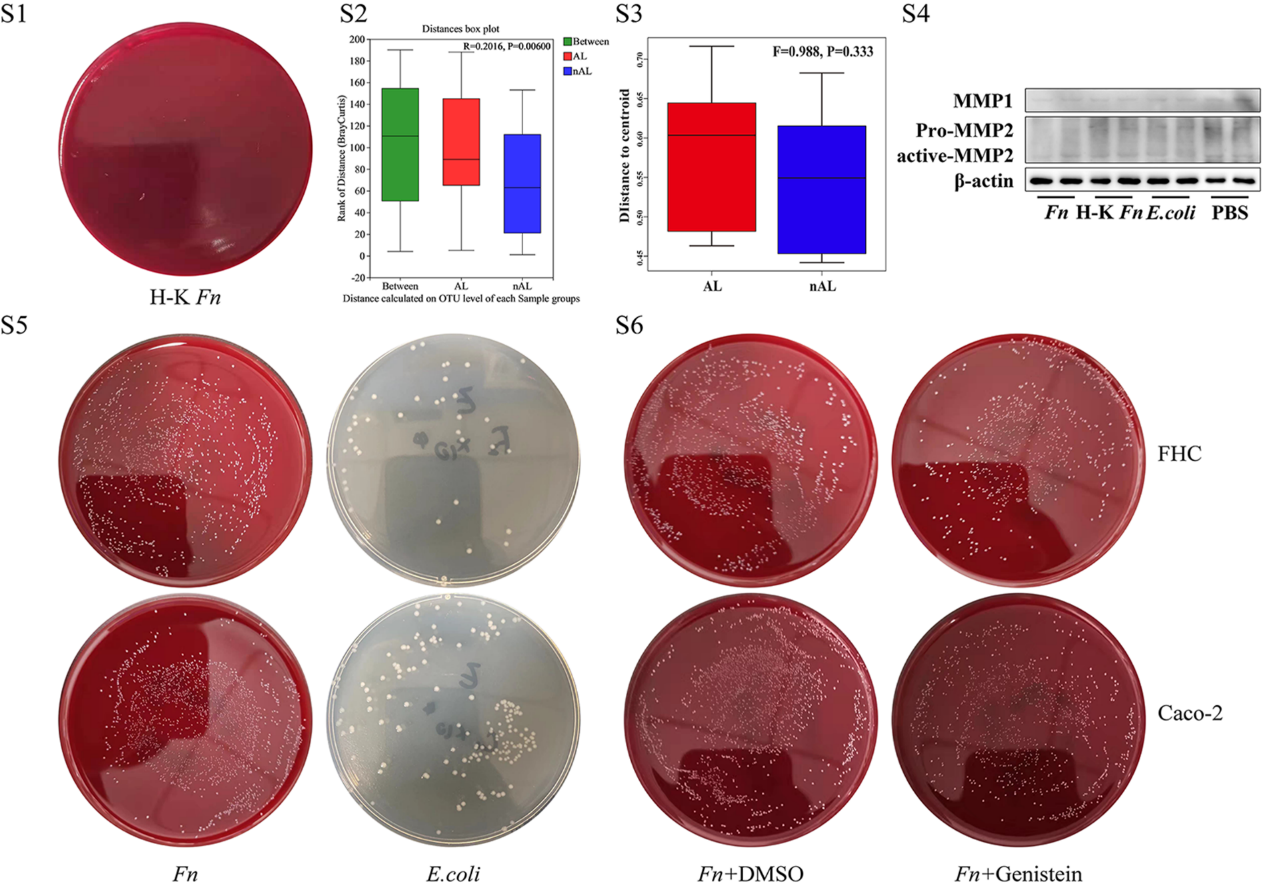


Figure S1. The blood agar plate inoculated with heat-killed *Fn.*

Figure S2. The ANOSIM analyses based on BrayCurtis distance.

Figure S3. The betadisper analyses based on BrayCurtis distance.

Figure S4. The protein expression level of MMP1 and MMP2 in anastomotic tissues.

Figure S5. The amount of attached *Fn* and *E.coli*.

Figure S6. The amount of attached *Fn* after genistein treatment.

1. Jin X, Liu Y, Yan W, Shi S, Liu L, Lin B, Guo X, Cai T, Wei Y: **Gut microbiota from nCAL patients promotes colon anastomotic healing by inducing collagen synthesis in epithelial cells**. *Journal of gastroenterology and hepatology* 2022, **37**(9):1756-1767.

2. Arthur J, Perez-Chanona E, Mühlbauer M, Tomkovich S, Uronis J, Fan T, Campbell B, Abujamel T, Dogan B, Rogers A *et al*: **Intestinal inflammation targets cancer-inducing activity of the microbiota**. *Science (New York, NY)* 2012, **338**(6103):120-123.

3. Chen S, Su T, Zhang Y, Lee A, He J, Ge Q, Wang L, Si J, Zhuo W, Wang L: **Fusobacterium nucleatum promotes colorectal cancer metastasis by modulating /KRT7**. *Gut microbes* 2020, **11**(3):511-525.

4. Castellarin M, Warren R, Freeman J, Dreolini L, Krzywinski M, Strauss J, Barnes R, Watson P, Allen-Vercoe E, Moore R *et al*: **Fusobacterium nucleatum infection is prevalent in human colorectal carcinoma**. *Genome research* 2012, **22**(2):299-306.

5. Wang T, Sun X, Cui H, Liu K, Zhao J: **The peptide compound urantide regulates collagen metabolism in atherosclerotic rat hearts and inhibits the JAK2/STAT3 pathway**. *Molecular medicine reports* 2020, **21**(3):1097-1106.

6. Wołosowicz M, Łukaszuk B, Kasacka I, Chabowski A: **Diverse Impact of N-Acetylcysteine or Alpha-Lipoic Acid Supplementation during High-Fat Diet Regime on Matrix Metalloproteinase-2 and Matrix Metalloproteinase-9 in Visceral and Subcutaneous Adipose Tissue**. *Cellular physiology and biochemistry : international journal of experimental cellular physiology, biochemistry, and pharmacology* 2022, **56**(2):166-179.
